# The Online Bingo Boom in the UK: A Qualitative Examination of Its Appeal

**DOI:** 10.1371/journal.pone.0154763

**Published:** 2016-05-03

**Authors:** Martine Stead, Fiona Dobbie, Kathryn Angus, Richard I. Purves, Gerda Reith, Laura Macdonald

**Affiliations:** 1 Institute for Social Marketing, School of Health Sciences, University of Stirling, Stirling, United Kingdom; 2 School of Social and Political Sciences, University of Glasgow, Glasgow, United Kingdom; Tianjin University of Technology, CHINA

## Abstract

Online bingo has seen significant growth in recent years. This study sought to increase understanding of this growth by exploring the appeal of online bingo. Our aim was to examine the content of ten online bingo websites in the UK and analyse a qualitative secondary dataset of 12 female bingo players to investigate the appeal of online bingo. Using two distinct data sources allowed us to assess how the key messages online websites are trying to convey compare with actual players’ motivation to play bingo. Our analysis of bingo websites found a common theme where websites were easy to navigate and structured to present a light-hearted, fun, reassuring, social image of gambling. In addition, the design decisions reflected in the bingo sites had the effect of positioning online bingo as a benign, child-like, homely, women-friendly, social activity. Comparison of the website content with our participants’ reasons to play bingo showed congruence between the strategies used by the bingo websites and the motivations of bingo players themselves and the benefits which they seek; suggesting that bingo websites strive to replicate and update the sociability of traditional bingo halls. Online bingo differs from traditional forms of bingo in its ability to be played anywhere and at any time, and its capacity to offer a deeply immersive experience. The potential for this type of online immersion in gambling to lead to harm is only just being investigated and further research is required to understand how the industry is regulated, as well as the effects of online bingo on individual gambling ‘careers.’

## Introduction

Bingo is a game of chance, played in a licensed club/hall or an online licensed site (‘remote’ bingo), in which players purchase cards covered in numbers which are marked off as the numbers are drawn from random. The player who marks all the numbers first wins a prize, similar to the games keno and lotto. Bingo is increasingly popular all over the world, with an online bingo market in the European Union estimated at approximately €926.6m in gross gaming revenue in 2013 [1]. The UK is the world’s largest single online bingo market, estimated at €312m of gross gaming revenue (GGR) in 2013 [1]. A particular feature of bingo in the UK is that it is the only form of gambling in which women engage more than men [2–6], and is consistently associated with lower socio-economic status and deprivation [2,6,7]. In the 2012 Scottish Health Survey, bingo was the most popular form of gambling among women after lottery participation and scratchcards, and was played by 10% of women [8]. The 2012 Health Survey for England reported that 7% of women played bingo (compared with 3% of men), but this increased to 12% of women in the most deprived IMD (Indices of Multiple Deprivation) quintile [9].

Prior to 2005, online bingo sites were subject to strict regulation in the UK concerning advertising. However, this changed after the introduction of the 2005 Gambling Act which dispensed with a number of advertising regulations and allowed online bingo operators to advertise on television, print media, cinema, outdoors and radio. A 2013 report on trends in UK television gambling advertising found that exposure to bingo advertising increased ten-fold between 2005 and 2012, with adults experiencing an increase in exposure from 1.2bn ‘impacts’ (the number of times an advert was seen by viewers) in 2005 to 12.7bn impacts in 2012, and children experiencing an increase from 87m to 723m impacts over the same period [10]. Data from the bingo industry confirms this sharp increase: for example, UK bingo provider Tombola increased its main media (print, outdoor, radio, cinema and television) advertising expenditure from £254,000 in 2011 to £2.72m in 2012 [11]. While traditional bingo halls and clubs have declined in popularity in the UK in recent years, reporting a drop in overall ticket sales of 14% and a drop in overall participation fees of 8% between 2009–10 and 2013–14, online bingo has proliferated [12]. UK turnover at remote bingo sites increased from £3.94m in 2008–09 to £44.40m in 2013–14 [12]. In 2004, there were fewer than 20 online bingo providers in the UK compared with more than 437 in 2015, although the market is fluid, with smaller operators constantly entering and departing the market [13]. The proliferation in online bingo is a phenomenon which has not been fully explored to date.

Online forms of gambling have many perceived benefits for both novice and regular gamblers. For example, they provide: an easy initiation for the novice, whereby they can play for points or free money until they have developed the experience and confidence to use their own money, and where the potential anxiety and embarrassment of entering a bricks-and-mortar establishment and having to master new rules are lessened [14,15]; offer almost unlimited opportunities to gamble, with no closing hours and no need to leave one’s own home [16,17]; and offer a vast variety of games [14]. In addition they are perceived as a good value for money form of entertainment; offer social contact and a sense of community [16], reduce isolation and boredom, particularly for those with limited real world social contact [18]; and offer a form of escape and release from the stresses of family life [19]. For women in particular, online gambling is seen as less of a male domain and a fun and safe environment where they can learn to gamble [14,20]. Female online gamblers have been found to prefer easy games, and to be attracted by features such as sound and graphics and large jackpots [16].

All of these features add up to a potentially highly engaging experience which delivers considerable emotional benefits. A market intelligence report on the gambling industry noted that, with online gambling, *“Consumers no longer need to leave the home to place bets in a retail outlet*.*… As such*, *internet gambling is potentially fuelling a greater ease for consumers to be drawn into the industry’s addictive nature as bets can be placed more impulsively at anytime*, *regardless of retail opening hours”* [3]. Insights into the potential harm arising from this unfettered online access are provided by studies conducted with online gamblers and problem gamblers, including online bingo players. These illustrate how women’s engagement in an initially light-hearted social activity can develop into a more and more compulsive behaviour, associated with withdrawal from family life and relationships, disrupted work and sleep patterns, and feelings of helplessness [18,19].

The number of people classified as exhibiting problem gambling behaviour and experiencing harm through online gambling is low and the proportion of harm linked specifically to bingo is likely to be even lower [9]. However, it is possible that the effects of the recent proliferation in online gambling will only emerge in future years. Statistics published by the UK gambling addiction charity GamCare reported that the second most cited location for problem gambling behaviour was the online gambling sector (34% of calls to the helpline) [21]. Although it is unclear how much of this is related to online bingo, evidence is emerging to suggest that online gambling participation in the UK is increasing faster among women than men [16].

To date, there have been no published studies which focus on understanding the growth and appeal of the phenomenon of online bingo websites in the UK. This study sought to fill this gap. Our study aims were to:

- Identify and analyse in detail the characteristics of online bingo
- Explain the potential appeal of online bingo in the UK to bingo players

This study describes and analyses the thematic content of online bingo websites in order to explore how they seek to attract, engage and influence their target audiences. We approached this from two distinct angles. First, we conducted a content analysis of online bingo providers’ websites to identify the strategies used to appeal to new and current players. Thematic content analysis is a well established and widely recognised research method for examining texts, media and online content, and is particularly suited for describing new media phenomena and for understanding and analysing the message strategies and framings deployed on a particular topic [22–24], including online gambling [25]. We then conducted secondary analysis of an existing longitudinal qualitative dataset of gamblers who played bingo (in bingo hall and online) to understand their motives for playing bingo. This approach allowed us to explore how participants’ accounts of their behaviour and motivations compared with the key messages online websites are trying to convey to encourage bingo players to play online. Using two separate and distinct data sources added greater depth and insight to investigate our hypothesis, which was that online bingo websites would use design, content and other features to offer a distinct form of immersive gambling which appeals to these motives and attracts bingo players to play online.

## Methods

### (i) Analysis of websites

A content analysis (where elements of a set of documents are systematically recorded for examination) was conducted on 230 webpages from ten bingo websites in the UK: 32Red Bingo, Cheeky Bingo, Costa Bingo, Dream Bingo, Foxy Bingo, Gala Bingo, Ladbrokes Bingo, Mirror Bingo, Paddy Power Bingo and Tombola. It was hypothesised that a player new to online bingo would use a search engine to find a website. Thus the sites were selected using Google.co.uk to search for the term ‘bingo’ on one date in May 2013, and the first ten hits for bingo websites selected. (Cookies were deleted before running the search to attempt a ‘neutral’ search, unaffected by previous searches.) This sampling procedure provided a range of types of online bingo companies available in the UK: some of the companies also operated bricks-and-mortar bingo halls (eg. Gala Bingo); others were newer, online only bingo companies (eg. Cheeky Bingo and Tombola); some were bingo websites developed by betting and gaming companies with a longstanding presence in the UK and Ireland (Ladbrokes Bingo and Paddy Power Bingo); and one represented a common brand sharing format in online bingo, named for an established print or broadcast media company (Mirror Bingo, named for the Daily Mirror, a UK tabloid newspaper). Most were based in offshore jurisdictions (eight in Gibraltar and one in the Republic of Malta).

Three stages were involved in the data capture and analysis. Firstly, the main content of each website was recorded using a structured protocol. A draft protocol was initially piloted by four authors (MS, KA, RIP, LM) on two websites (two authors per website), then refined and used to capture data from the ten bingo websites. The protocol comprised a form with tick box lists and some open-ended sections (see S1 File), plus an NVivo 10 (QSR International, Victoria, Australia) database to record images (screengrabs) of the webpages. Starting with the homepage, the presence of links, features and information were noted in the form and an image of the full page captured; then the links to the main sub-pages of the website were clicked on, captured for NVivo, and again, the presence of pre-determined links, features and information identified on the form. Open-ended sections allowed other items or features to be captured, and websites could be browsed using site-maps (where available) to track down information for the protocol that was not directly linked to from the homepage or main subpages. The data were input to Excel and summarised in descriptive tables.

Secondly, the extent to which the site could be accessed by a new user was assessed. This involved exploring which areas of the site could be accessed without registration, and then assessing the registration process by creating a pseudonymous customer account using a standard name, email address and date of birth. We did not enter credit card details into any of the sites but did assess how much of the site content could be accessed without doing so (for example, whether it was possible to play without adding credit card details).

Thirdly, a detailed thematic analysis was conducted of the imagery, design and ‘message appeals’ of all 230 pages. In marketing and advertising communications, ‘appeals’ are the persuasive strategy employed in the message, or the “selling message” [26]. Images of the webpages collected at the first stage were entered into a database for coding, using the qualitative data analysis software NVivo 10 [27]. Each page was coded first by company and page descriptor (eg. ‘Paddy Power, Home page’). Pages were ‘read’ by members of the research team and five initial coding categories were identified to represent key elements of the page (colours, imagery, typography, textual and overall design features), and four initial coding categories for messages appeals (e.g. appeals around ‘recruitment and relationships’, ‘play and winning’, ‘frequent and constant play’ and ‘loyalty’). Themes were then identified within each category through further reading and discussion, and additional categories and themes added for a final thematic coding frame of ten overall coding categories and 68 themes (see S2 File). Visual and textual elements on each page were coded and entered into framework matrices. The coding frame was initially piloted by three authors (MS, KA, RIP) on two bingo sites (two authors per website) then refined. Answers were compared and discussed to improve coding reliability for the rest of the websites which were all singly-coded by three authors.

### (ii) Secondary analysis of in-depth interviews

The website content analysis explored the design of bingo websites focusing on their appeal to attract new players and build loyalty, but could not tell us why people played bingo. Thus, secondary analysis of interviews from 12 bingo players augmented findings by exploring motivation to play bingo from the perspective of initiation and continued play. These interviewers were part of a five year, longitudinal, qualitative, cohort study of 50 gamblers called *‘Situating Problem Gambling’*. For more information on this study see Reith and Dobbie [28,29].

All 12 bingo players were female with a mean age of 50 years, (age ranged from 20 to 73 years). Employment status varied with older participants tending to be retired and the remainder either in paid unskilled employment or unemployed. Interviews took place at a time and place convenient to the participant and were conducted by two authors (FD, GR) and four highly experienced, female, qualitative interviewers. Ethical approval was granted from the National Centre for Social Research and participants gave written informed consent. Participants were recruited from Glasgow, Scotland and unknown to interviewers prior to study commencement. Two recruitment methods were used ‒ face to face from a bingo hall, casino, bookmaker and advertising in the local press and community venues (libraries, health centres, community centres). Interviews took between 30 and 90 minutes, were digitally recorded, transcribed and field notes written after each interview. A thematic analysis was conducted to systematically code, classify and organise interview content into key themes. The first stage of analysis was to make sense of the data through a process of transcript familiarisation, sorting and coding. Two authors (FD, GR) developed a coding frame and data were coded by all interviewers using Framework [30]. This created a platform for movement beyond what Braun and Clarke refer to as the *‘surface of the data’* (i.e. descriptive analysis), and into interpretive analysis [31].

## Results

### (i) Overview of websites and access procedures

The bingo websites were easy to navigate and generally used the same structure of an opening homepage linking to subpages for joining and registration; playing bingo and other games; community features (eg. live chat features or a players’ forum); promotions and offers; a loyalty scheme; links to other promotional channels (eg. social media sites or television adverts); and information on regulations, safety and the company. Table 1 summarises key aspects of the websites’ content, and shows that most offered a wide variety of games, promotions and other services. Nine of the sites encouraged play immediately, on the Home page, and most linked on the Home page to promotional offers. Promotions included big prize money, new member promotions and free games. Nine of the ten sites offered other forms of gambling, most commonly slots and casino games (7 sites each). Three had a Community chat forum, and all ten had a loyalty scheme, with tiered levels of membership in three of the sites. Six sites linked to a mobile app and seven to social media. Six offered a live chat customer service facility. All sites had some information about self-exclusion, although this was flagged up on the Home page on only four of the sites.

**Table 1:** Content featured in the ten bingo websites analysed.

Content	Number of websites
Encouragement to ‘play now’ on Home page	9
Promotional offers on Home page	8
Types of promotions offered:	
*Big prize money*	10
*New member promotions*	9
*Free games*	8
*Bonus/cash gift on joining*	5
*Discounted/low game fees*	4
Other forms of gambling offered:	9
*Slots*	*7*
*Casino*	*7*
*Scratchcards*	*5*
*Poker*	*3*
*Sports betting*	*3*
*Lotteries*	*2*
Community chat forum	3
Loyalty scheme	10
Live chat customer service	6
Link to mobile app	6
Link to social media (eg. Facebook)	7
Responsibility content:	
*Responsibility logo on Home page*	7
*Information on self-exclusion on Home page*	4
*Information on self-exclusion on other page*	7

All but one of the sites required a new user to register (one, Foxy Bingo, allowed the use of the community features by registering through Facebook rather than registering an account). However, the registration process was relatively straightforward for all sites, presenting no obstacles which would be unfamiliar to people used to shopping online, and taking between five and ten minutes; Costa Bingo promised ‘you’re only 2 minutes and one simple form away from joining and starting the bingo fun’. All ten sites asked for date of birth, and it was not possible to enter a date of birth under 18 years of age on any of the websites, but only four websites included additional age verification procedures (namely, a box which the user checked to confirm they were over 18). None of the websites ran checks or verification procedures for personal contact details (address, phone number, email address) before completing registration. Eight of the ten websites offered registration bonuses for those opening an account (for example, a ‘free’ £5 play bonus).

Three websites allowed users to play games without entering credit card details as part of the registration process, and five websites allowed users to play games without depositing funds. Cash prizes were offered for free games on four out of the ten websites, meaning that new users could start to win before they had registered their credit card details with the site, and acting as an enticement to continue playing.

### (ii) Thematic analysis of bingo websites

The thematic analysis examined how design, imagery, textual style and content were used in conjunction with each other to engage both new and existing users and how the benefits of online bingo were conveyed. Findings have been combined into three over-arching themes: drawing in the first-time user, creating belonging, and stepping up involvement. Table 2 illustrates the main themes with examples of findings.

**Table 2:** Themes in the qualitative content analysis of online bingo sites.

Overarching theme	Sub-theme	Example
**1. Drawing in the first time user**	Online bingo is exciting	Use of design features to convey excitement: eye-catching colours, block capitals, exclamation marks, images of gold, money
	Playing online bingo is normal and popular	Photos of other players
		Use of ‘feminine’ colours and references
		Friendly, unsophisticated design
	Winning is easy	Messages that there are winners every day, every week
		Testimonials from other winners
	Access is easy for new players	Ability to play games without depositing money
		Tips on how to play and how to understand ‘bingo lingo’
**2. Creating belonging**	Online bingo offers social interaction, community, friendship	Inviting, inclusive language
		Profiles of chatroom hosts/moderators
		Players invited to share stories
	Online bingo replicates some of the social features of traditional bingo halls	‘Chat’ and ‘Community’ sections
		Ability to play games while chatting
		Scheduled events such as online ‘coffee mornings’
**3. Stepping up involvement**	Online bingo can be part of everyday life	Encouragement to play bingo 24/7, round the clock, while on the go
	Online bingo offers variety and progression	Variety of different bingo games
		Encouragement to play other games (eg. online slots) between bingo
	Greater rewards from greater involvement	Loyalty schemes offering prizes or other rewards for players who spend a specific amount or play for a specific length of time
		Different loyalty tiers, with better rewards for higher spenders

#### Drawing in the first-time user

For the first-time visitor, the sites presented the prospect of an exciting, likeable and easily accessible experience. There was a pervasive feeling of breathless excitement and action conveyed through textual injunctives–“join now”, “play now”, “win now”–and busy, clashing designs which gave the impression of constant activity. The sites evoked the thrill of playing and winning through constant mentions of the prize money that could be won, and through lists of or testimonials from prize winners, sometimes constantly updating on the Home page. The prospect of easy wins and fantastic rewards were held out enticingly: ‘life changing wins’ [32Red Bingo]; ‘top treats’, ‘ravishing rewards’, ‘major millions’, ‘mega moolah’, ‘where all your bingo wishes come true!’ [Dream Bingo]; ‘10,000 winners every week’ [Ladbrokes Bingo]; ‘15,000 [promotions] to be won every night’ [Paddy Power Bingo]; ‘Vegas Vacation’ prize [Gala Bingo]; and ‘£5 million won every week” [Tombola].

At the same time as conveying this impression of thrilling activity and transformative wins, the sites also presented a reassuring image of bingo as normal, widespread and everyday. Bingo was presented as popular and ubiquitous, with ‘thousands of players’. Photographs of chat hosts and winners, used on nine of the sites, were often low quality, snapshot style of the sort that users themselves might take, rather than glossy studio shots, potentially encouraging identification and providing reassurance. Although the sites did not speak exclusively to women—male hosts and winners were featured on several sites—the impression was that the sites were designed largely to appeal to women, through use of the colours pink and purple, images of hearts, cocktails, fashion and glitter balls, offers on beauty products, and references to ‘mums’ (the Foxy site was associated with the Rugby Football League ‘Mum of the Year’ award, and featured nominations from users).

The impression of bingo as a normal, everyday activity for fun-loving women was further underlined by a particular design style used across the majority of the sites. This style was characterised by a use of design elements evoking child-like fun rather than adult-oriented sophistication: vibrant and unsubtle colours deployed in cartoonish combinations such as yellow and blue (Costa Bingo), purple and orange (Foxy Bingo) and pink and yellow (Cheeky); typography redolent of children’s comics or down-market magazines, with frequent use of block capitals, often in clashing fonts, and call-outs; and imagery primarily comprised of clip-art images, such as graphics of gold coins and crude line drawings. The unsophistication of the sites’ visual appearance was echoed in their use of language, with frequent use of silly, alliterative wordplay (eg. ‘consolation station’, ‘double trouble’, ‘fabby fun’, ‘bingo bonanza’ and ‘bouncy balls) and demotic speech (‘spreading the lurve’, ‘mega moolah’), again contributing to an impression of bingo as a lighthearted, normal and unthreatening activity. Two further aspects of this design style were notable; firstly, that where the bingo provider was better known for its other gambling services (eg. Ladbrokes and Paddy Power), its other websites did not use this style, and secondly that of the pair of sites which used more muted versions of this design style, both were re-designed after the fieldwork to come closer to the norm.

The new user was enticed into play through a range of strategies. Five of the sites made offers of ‘free play’, to entice users into sampling the games, and eight offered ‘free’ money to new players, such as Foxy Bingo’s offer of a ‘free’ £20 on top of a £10 deposit”. Testimonials gave the impression that winning was easy: on the Mirror Bingo site, one winner described how it was ‘fantastic to win so soon after joining’. Throughout all the sites, constant reassurance was provided, with users being directed to instructions on how to join the site, how to play specific games, guides to ‘bingo lingo’ and chatroom textual conventions, live Customer Service chat, and community forums and chat rooms where they could introduce themselves and ask advice. At the same time as providing reassurance that novice status was no barrier to enjoyment, acceptance and winning, the sites also consistently presented references to a deeper level of experience available only to those who are regulars (see ‘stepping up involvement’ below), thereby further enticing new users.

#### Creating belonging

Belonging was a major theme in the sites. The language was inviting and inclusive, with constant references to joining in, social interaction, community and friendship. Use of the first and second person (for example, “we’re always really happy to hear from you” [Tombola] and “we love it when our players win big” [Mirror Bingo]) enhanced the feeling of involvement. This was further reinforced by vocabulary associated with friendship and participation: new visitors were invited to ‘join our gang’ and urged that ‘bingo is a social thing, so get yourself involved ppl [people]!’, while fellow players were described as chat ‘roomies [room-mates]’, Dream Bingo invited new visitors on its Homepage to ‘get to know other roomies and our Cheery Chat Hosts’. 32Red Bingo offered play and chat in a ‘Friendship Lounge’, and invited players to join its ‘community’, as an ‘opportunity to meet life-long friends’. Testimonials on the Mirror Bingo site spoke to this social dimension, quoting players as saying ‘we have a good laugh’, ‘everyone is really friendly’ and ‘a great way of socializing, as I can’t go out a lot’.

‘Chat’ and ‘Community’ pages were major features of the sites, offering the facility to chat with ‘chat hosts’ or ‘room hosts’ and other players during and between games. Some sites included lists of bingo and chatroom terminology for new users (e.g. ‘BRB’—be right back, ‘GOOD4U’—good for you [32Red Bingo], ‘WDW’—well done winner(s) [Tombola]). Chat hosts and room hosts were often presented as real people: for example, on four sites, each host was introduced with a photo (Foxy Bingo and Gala Bingo) or avatar (23Red Bingo and Tombola) and a description seemingly in the hosts’ own words. These features helped to give a personality to the brand to which users could relate. Four of the sites featured a brand mascot or icon—the Costa Bingo sun, called ‘Sunny’, Cheeky Bingo’s ‘Cheeky’, a cartoon young woman with exaggerated facial features, Foxy Bingo, an anthropomorphised fox in a suit, and 32Red Bingo’s anthropomorphised red, number 32 bingo ball. The mascot/brand figure/icon appeared on every page and often ‘spoke’ to the site user in the first person–“my little Cheekies”, “my Foxy friends”–again helping to build a relationship between brand and users. Users reciprocated this convention, with winner testimonials on the Foxy Bingo site addressing their gratitude to the mascot and the brand: “Thanks so much Foxy. First big win so I was over the moon when number 87 came out, I was shaking I was that happy!”; “I’m spending my winnings on a nice holiday with my daughter somewhere hot!!! Thank you so much Foxy!!!”. The Foxy Bingo site also had its own magazine, FoxyFriends, offering ‘all the gossip’, and invited user nominations for the ‘Rugby Football League Mum of the Year’ competition. These involved users sharing personal stories about their family lives, and could be seen as encouraging a deeper emotional engagement with the site and with other users.

Overall, these community and chat features can be seen as attempting to replicate the social experience of traditional bingo halls: 32Red Bingo described itself as ‘the internet’s friendliest online bingo hall’, Foxy Bingo described its regular Monday to Wednesday morning games as ‘Coffee Mornings’, and Paddy Power Bingo urged visitors, ‘You make the tea, we’ll bring the fun’. They can also be seen as features intended to inject interest and variety into users’ play experience, encouraging longer and more frequent visits to the site.

Perhaps most importantly, in a market with considerable fluidity, the sites could be seen as aiming to create feelings of belonging which would create and cement relationships between the user and the game, other users, and ultimately the brand. A good example of this was the way in which existing players were incentivised to act as recruiters, with offers of bonuses (£15 on Costa Bingo, £20 on Dream Bingo) for signing up a friend. As well as being a cost-effective way of drawing in new customers, the strategy of getting friends to involve friends could be seen as helping to cement users’ relationship with the site and with embedding bingo further into their everyday life.

#### Stepping up involvement

The sites used a number of strategies to encourage continuing and deeper involvement. To retain users’ interest and to replicate the wide range of play modes found in traditional bingo halls, a wide variety of bingo games were offered, at different prices and with different play and win modes (eg. 90, 80 and 75 ball bingo; multiple card options; wins for a line, 2 lines, a full house or a shape/pattern on the card). The Ladbrokes Bingo site stated ‘If you thought there was only one way to play bingo then you’re in for a treat’ while Tombola promised games which ‘cater for everyone’s budget’ and ‘something to suit everyone’ and asked ‘which will be your favourite?’

Users were encouraged to embed bingo into their daily routines: Gala Bingo offered a Saturday Night Takeaway game, Mirror Bingo a Sunday Night Jackpot Race, and Tombola a ‘breakfast bingo’ promotion. On the Mirror Bingo site, one user testimonial describes regular early morning play as ‘a good laugh which sets me up for the day to tackle my job’. The potential to play frequently and continuously was emphasised: Costa Bingo promised games ‘every three minutes’ and promotions ‘24 hrs a day’, while one section of Ladbroke Bingo’s site listed a schedule of games starting just 15 seconds apart. Users were encouraged not only to play ‘now’ but also to build play into the next few weeks: Dream Bingo, for example, invited users to play specific slots on specific dates up to a month ahead ‘to earn doubled Loyalty Points’. Several sites offered the facility to ‘pre-purchase’ tickets for future games, ‘where you can make sure you’re still in with a chance to win, even if you’re not around on the night for the big money games’ [Mirror Bingo]. Several sites made explicit appeal to a ‘fear of missing out’, exhorting players ‘don’t miss out’, play today’ and ‘what are you waiting for’ [Ladbrokes Bingo], and, for a mobile bingo app, ‘no more missing out’ now that users could ‘play on the go’ [Foxy Bingo]. Metaphors of achievement and accumulation throughout the sites–‘conquer Money Mountain’, ‘earn enough points’ [Dream Bingo], ‘join our board of winners’ [Tombola]–further reinforced engagement.

Furthermore, users were encouraged to stay engaged and to play *between* bingo games–‘keep winning while you wait for your next Bingo game to start’ [32Red Bingo]–with encouragements to play slots, scratchcards, lotto, card games, casino games and others. Some offered chat games to play whilst in the community/chat forums, including word games and trivia quizzes; on the 32Red Bingo site, users could play chat games simultaneously linked to their bingo play, such as typing words linked to the bingo calls. The language used on the Ladbrokes Bingo site hinted at dependence, with exhortations to ‘Fill the gap while you wait’ between bingo by playing other games, and presenting ‘mini games’ as a way to ‘cure the itch’. Dream Bingo also encouraged chasing losses, with a ‘cool Consolation Prize Free Spins’.

Another key strategy for deepening users’ involvement was to link rewards to engagement, in particular by incentivising frequent play or high spend. Mirror Bingo offered ‘The More you Play Tournament’ in which the three players to purchase the most bingo cards during the previous week-long period would win additional Bingo Points (which could be converted into cash to purchase more bingo cards). Dream Bingo offered a Wednesday ‘Goodie Grab’, in which users had to play ‘for at least an hour and a half to be eligible for prizes’. Loyalty schemes were structured so as to reward more activity with more points, bonuses or play money, and three of the sites classified scheme members into different loyalty tiers on the basis of spend; for example, Gala Bingo, with a five-tier scheme, promised ‘the higher your level, the more BuzzPoints you’ll earn each time you play!’. One of the most complex schemes was that on Dream Bingo’s site, which offered six tiers, through which users could progress once they had earned enough to meet the next threshold. Users then had to play and spend at a target level in order to maintain their tier position. Additional loyalty points were awarded on an increasing scale for movement up the tiers (eg. 10% extra points for attaining Silver, 20% for attaining Gold, through Platinum, Diamond and ‘Privé’, which earned 60% extra loyalty points). Points could be redeemed for Casino Bonuses, with extra levels of bonus being awarded for redeeming a higher number of points. The Foxy Bingo site tantalised users with an ‘Elite Club’, in which members receive ‘personal attention from our devoted account Club Manager’, ‘the largest bonuses’, ‘real cash prizes’ and ‘luxurious gifts personally selected’; conditions for membership of the club were shrouded in mystery (‘by invite only’; ‘so exclusive that the finer details are kept a secret’).

As discussed in the Introduction, research with bingo players reports that frequent players describe struggling with feelings of guilt about the amount of time and money they spend on bingo sites, sometimes contrasting it with the time or money they could have spent with their families instead [18,19,28,29]. As if to acknowledge and neutralise this concern, the sites made reference to families and mothers, often in the context of showing what treats could be provided for the family through winning. On one site’s homepage, a mother described how she would ‘spend the jackpot on my beautiful girls’ (Tombola); on Costa Bingo’s homepage, it announced “giving away top treats every day this week in our Doting Dads bingo games, giving you the perfect gifts for Father’s Day!”; and there was Foxy Bingo’s association with the Rugby Football League ‘Mum of the Year’ award, described above.

### (iii) Secondary analysis of qualitative interviews

#### Bingo players’ motivations to play bingo

Analysis of transcripts collected from 12 bingo players highlighted similar motivations for playing bingo as those the websites are trying to convey. In addition, this analysis also adds context around motivations to play bingo and potential risk factors associated with playing bingo. Table 3 presents the main themes and sub-themes with examples of coded text. Findings are presented around three main themes: initiation into playing bingo; on-line bingo appeal (case study of one participant); variety in bingo games and other types of gambling.

**Table 3:** Themes and coding examples for qualitative interviews with bingo players.

Overarching theme	Sub-theme and coding instructions	Sample of coded text
Initiation into playing bingo	The first time they heard of bingo or knew of someone who played bingo.	Remembers when she was 14 going down to meet her mother at Gala bingo.
	Family and friends history of playing bingo	Mother went to bingo, had uncles that gambled on horses, father wasn't a gambler, only gambled on a big race like Grand National
	Their first bingo experience	First time she played bingo was at the Old Vogue with her mum aged 18.
On-line bingo appeal	Frequency and duration of play	She hasn't been to bingo for 6 months, but now plays online bingo. Has done so for 4 months.
	Reasons for playing/likes and dislikes of playing	Likes online bingo because if you play with £10 they give you an additional £5 to play with.
	Strategy for playing	She has her shower, moisturises, puts a face pack on, fresh pyjamas, has a glass of wine, a smoke & plays bingo online. She sits & chats with the others.
Variety in bingo games and other types of gambling.	Type of gambling	She'd heard about the online bingo from a couple of women at the bingo. She recently tried a different online bingo run by the Sunday Mail. She got £10 free without having to put any money in herself. She played with that £10 & has not been on since as didn't like it. Found it tacky & less friendly than the STV one she is used to. (13)

A common theme around initiation was the role of a ‘facilitator’, typically a female relative or friend whose evident enjoyment of the game encouraged the participant to want to try it for herself. One participant recalled as a child going to meet her mother at the local bingo hall. Standing outside waiting for her she would listen to bingo caller, absorb the atmosphere and feel the camaraderie amongst the players.

*“You used to see her [her mother] coming out of the bingo and she used to have a carry on [a laugh] with the staff …*. *I think it was just standing outside*, *hearing the caller and things like that*, *that I just…it was quite…it was the atmosphere of the people coming out as well do you know what I mean*, *it looked quite enjoyable and that’s when I just thought ‘I’ll give it a go’*.*”*

(Female, aged 29 years)

As this is a secondary analysis of an existing dataset, primarily focusing on bingo played in bingo halls, we cannot be sure whether a similar pattern exists for initiation to online bingo—i.e. whether online players are introduced by someone else or not. However, the website analysis highlighted the importance of ‘recruiters’ to bring in new members which was encouraged via financial reward. Thus, the role of a ‘facilitator’ is found in online bingo as well as traditional bingo halls, albeit with a slightly different focus.

A key theme from the website analysis was the strategies used by bingo websites to convey a sense of community and friendship. This was also found in individual narratives for both initiation and continued play (discussed below). An example comes from one participant who described how her sister introduced her to bingo after her partner died as way to get her out of the house and socialise with other people.

*“And then because I wasn’t getting out much*, *two years ago my sister said come on you will need to start doing something because I don’t drink or anything*. *I don’t go to pubs or anything*. *So I started going out like once a month with my sister and then I met Tina [bingo friend] two years ago and I started going down for the company basically*.*”*

(Female, aged 50 *years)*

Of particular interest was one participant who, over the course of the fieldwork, switched from playing in a traditional bingo hall to playing exclusively online. Helen’s reasons for preferring to play bingo on line were four-fold and resonate strongly with the key thematic messages of online recruitment.

First was value for money. Helen felt that her money lasted longer online than it would in a traditional bingo hall and she was therefore saving money. Also, if Helen played after midnight she felt there was a better chance of winning because there were fewer people playing. However, she also commented that the jackpots were smaller online in comparison to a traditional bingo hall (which is interesting because website analysis found a key content feature was the ‘big prize money’) but, for her, the thrill was simply playing bingo so this was an easy trade-off.

Second was the flexibility and variety that online bingo offered. Helen liked the fact that online bingo could be played 24 hours a day which meant she could play whenever she wanted to (unlike traditional bingo halls that have set opening hours). As was found in the website content analysis, Helen also commented on the variety of games that online bingo offered and liked the option of switching games if she was not winning.

Third was ‘me time’. Helen liked to play on a Friday night to ‘make a night of it’ and ‘pamper’ herself and enjoy a bottle of wine and smoke cigarettes whilst playing bingo online.

*“I have my Friday night ritual*. *I have my shower*, *I moisturise my body*, *I give myself a face pack*. *I do all these things on a Friday*, *night get my bottle of wine and I come in and I sit there*, *my cigarettes my bottle of wine and my bingo and I sit and chat and I love it*. *And maybe it you know I could go on that and say maybe 8 o’clock at night I could be on to it until 1 or 2 in the morning*, *I’ve only spent 20 pound*.*”*

(Female, aged 60 years)

Fourth, was social contact. Helen liked that she could have a ‘chat’ with other women ‘*like myself’*, across the world.

*“It’s absolutely amazing I was talking to [an online player] and do you know where she lives*, *Philadelphia and she is on so that’s the kind of thing I mean I get a real buzz out of it*.*”*

(Female, aged 60 years)

Despite being fully aware that the women she spoke to may be completely different in real life, Helen enjoyed the anonymity that playing online offered. For example, she liked the fact that she could make up a user name, that the people she communicated with did not know her real name, and that she could decide how much personal information she wanted to share.

In addition to online bingo, participants also cited a variety of ways to play bingo in a traditional bingo hall: pen and paper (i.e. a bingo card / book and pen to mark up numbers); using a hand held electronic device which allows the player to play multiple games of bingo and one time; mini bingo; national bingo; bingo whiz; party link bingo; and party boards. This variety was also a feature of bingo websites, which not only offered a range of bingo games but also different types of gaming (online slots, casino games, scratchcards etc., see Table 1). This may could that websites are trying to maintain interest and encourage longer periods of play which may result in members spending more time and money than planned or can afford. For example, in the transcripts, bingo players described how it was common to play other types of bingo (eg. mini bingo) in between the main games of bingo which became expensive.

*“Interviewer*: *So when you say ‘expensive’*, *what do you mean by that*?

*Participant*: *Well for your*, *say you put your books and you’ve got what they called PETS [programmable electronic tickets] computerised bingo right and I’ll have the PETS*. *You get more books for it you know but they do a deal if you buy the ehm*, *so I used to play the PETS*. *But it was like now how much was it for the morning*? *It was about 15 pounds but then your afternoon session with your lights and everything like that was about 22 so you take it that’s 37 pounds right*. *Before you do anything before you play mini bingo and you can lose another 20*, *30 pound on that*, *easy right*.*”*

(Female, aged 60 years)

Regular bingo players (i.e. those who played morning, afternoon and/or evening games) appeared especially vulnerable to playing machines, which could lead to a period of problem gambling. One participant spoke of playing on a machine to ‘pass the time’ in between games of bingo, but over the course of the fieldwork the amount of money she spent on machines quickly overtook that which she spent on games of bingo.

*“It’s [playing on machines] actually getting worse basically*, *you know…*.*once you’ve put quite a wee bit a money in*, *like money in the fruit machines…*.*like after you put 500 in*, *you start chasing to try and get something back and it didn’t happen*. *And it went to 1000*, *went to 1500*, *went to 2000*, *and still no return*, *you know*?*”*

(Female, aged 48 years)

In contrast, other players described how they had developed strategies to control the amount of money they spent on machines in between bingo games—e.g. listening to music on a mobile phone during breaks to distract them from playing machines.

## Discussion

Online bingo has seen significant growth in recent years. This study sought to increase understanding of this growth by exploring the appeal of online bingo, through thematic content analysis of online bingo websites in the UK and analysis of a qualitative secondary dataset derived from interviews with gamblers.

This study has limitations. The samples for both strands of investigation were small with one online case study. However, qualitative samples are not designed to be representative but to reflect range and diversity to explore and unpack the area of interest [30]. Thus, inclusion of the secondary qualitative dataset added value to the website content analysis. Future research using social network analysis [32] to examine the network structure, interpersonal connectivity and interactions of online bingo players may increase understanding of the appeal of online bingo.

Although the website sample was small (ten sites), each site contained large numbers of pages, which were reviewed in detail (230 pages in total). In addition, the UK websites selected may not necessarily be representative of bingo sites in other countries. Further investigation is, therefore, required to corroborate our findings in a European and international context. Finally, most of the website pages were coded by one of three single-coders in the content analysis and this entails a greater likelihood of bias than double-coding and comparing the results. However, the three single-coders had developed and refined the coding frame together, and they discussed any subsequent content they encountered that was difficult to code, all in an effort to maintain coding consistency.

Our study found that websites deployed a number of structural, textual and design features to draw in first time users. Sites were easy to access, with minimal age-verification procedures, and it was possible to play and win for ‘free’ even before entering credit card details. Textual and design elements evoked the thrill of playing and winning, while at the same time presenting a reassuring image of bingo as normal, widespread and everyday. Images, graphic design and references were strongly female-oriented and also designed to appear ‘unsophisticated’, suggesting a child-like, playful and benign activity. The design, colour, imagery, content of websites are often carefully considered, the result of decisions designed to meet strategic marketing objectives, based on congruence with desired brand image, intended target groups and consumer research (e.g. [33–35]). In our study, the design decisions reflected in the bingo sites had the effect of positioning online bingo as a benign, child-like, homely, women-friendly, social activity. This is in interesting contrast to other online gambling websites. For example, McMullan and Kervin’s [36] analysis of online poker sites found frequent use of adult-oriented imagery, such as young women in bikinis or adults depicted in sophisticated clothing and settings. The online poker sites in the McMullan and Kervin study were often highly international in outlook, emphasising their millions of players worldwide and offering multi-lingual content. The bingo sites in contrast were strongly regional and national in focus, emphasising their UK location (one, Tombola, made particular mention of its staff in Sunderland), and often used demotic and colloquial language, creating an impression of community and familiarity.

Belonging was a major theme in the bingo websites studied. Language was inviting and inclusive, with constant references to joining in, social interaction, community and friendship. ‘Chat’ and ‘Community’ pages offered the facility to chat with ‘chat hosts’ or ‘room hosts’ and other players during and between games. Mascots and other features were used to convey brand ‘personality’ and to build a relationship between brand and users. Features such as rewarding existing players to ‘recruit’ friends provided a means of drawing in new customers, cementing users’ relationship with the site, and embedding bingo further into users’ everyday life. The strategy of ‘customer referral’, using existing customers to draw in new ones from among their friends and family, is a widely used marketing strategy. Studies have shown that not only are such schemes an effective means of new customer acquisition, they can also increase existing customers' loyalty (ie. reduce the likelihood of their switching to another brand) and spend (eg.[37]).

Comparison of the website content with reasons women play bingo, from the secondary qualitative analysis, showed congruence between the strategies used by the bingo websites and the motivations of bingo players themselves and the benefits which they sought. The websites could be seen to be replicating and updating the sociability of traditional bingo halls. For example, for new users, the websites potentially replicate the role of the facilitator, the (usually female) friend or relative who demonstrates the appeal of bingo and introduces the user to the game. For existing users, the sites offer an apparently low cost, good value for money form of entertainment and relaxation which can be played anywhere. Although the face-to-face social contact of ‘bricks and mortar’ bingo is absent, community and chat features create a sociable experience which can be shared with others all over the world. Other research into the nature of women’s gambling and the benefits they seek from it supports these themes (e.g. [6]). Research in Sweden has suggested that women gamblers are more likely to participate in chance-based forms of gambling (such as bingo) in ‘domestic’ settings, and men to participate in forms of gambling involving strategy and in public settings [20]. An ethnographic study conducted with female gamblers in Canada found that gambling provided women with hope and escape, particularly against a backdrop of poverty and difficult lives, and particularly with an opportunity to socialise and a ‘sense of community’ which reduced their feelings of isolation [38]. Breen’s [39] qualitative study of older Australian bingo club players echoed these themes, observing that going to the bingo club enabled people who often felt isolated or bored at home to feel social in the company of others, and provided them with value for money entertainment and relaxation.

As well as using strategies with the potential to recruit new customers and convert them into regular visitors, the websites could be seen to deploy a range of strategies to encourage continuing and deeper involvement. These included: offering a wide variety of games to prevent boredom, encouraging users to embed bingo into their daily routines, emphasising the ability to play frequently and continuously, evoking the ‘fear of missing out’, providing the ability to ‘play on the go’, encouraging other forms of gambling between games, and linking rewards to engagement, in particular by incentivising frequent play or high spend. The marketing strategy of ‘cross-selling’–the selling of related services and products to existing customers—was used by nearly all of the bingo websites by offering other forms of gambling to the bingo players. For a company, this strategy aids customer retention and loyalty, hence maintaining or increasing sales. The qualitative interviews with bingo players hint at the potential for bingo playing to lead to problematic levels of involvement and to introduce players to other forms of gambling. Despite the portrayal of bingo as a relatively benign or ‘soft’ form of gambling, research has begun to suggest that, for some, play may escalate to problematic levels in ways similar to more traditionally ‘hard’ and / or masculine forms of gambling. This is the case with both online and offline forms. Furthermore, such escalation may occur in a manner that is particularly ‘hidden’, with women more likely conceal their playing from families and friends due to concerns about appropriate or respectable female behaviour, that revolve around concerns about stigma and shame [18,19].

Several areas of future research are suggested by the study. The first is to increase understanding of whether the recent proliferation in online bingo and online bingo marketing is associated with any corresponding increase in problem gambling behaviour and associated harm, particularly among women and lower income groups. Online bingo differs from traditional forms of bingo in its ability to be played anywhere and at any time, and its capacity to offer a deeply immersive experience. The potential for this type of online immersion in gambling to lead to harm is only just being explored. A recent qualitative study of 50 internet gamblers by Hing and colleagues [40] found preliminary evidence showing a link between online gambling advertising and promotional activity and an increase in gambling activity. Further research is needed specifically into the ‘gambling careers’ of online bingo players and into the relationships between online bingo marketing exposure, take-up of online bingo playing, and any progression into problem gambling behaviour.

Secondly, our study contributes to theoretical understanding of how gambling marketing strategies influence new and existing players. Our content analysis of online bingo websites suggested that the strategies used by websites performed three functions: drawing in new users, consolidating users’ relationship with the sites through creating feelings of belonging, and encouraging existing users to step up their involvement. In other words, website strategies could be seen as relating to initiation into gambling, reinforcement of gambling and escalation of gambling. Recent work in the addictions field has suggested that it is important to understand how the influences and determinants of substance use and gambling vary across these three stages of behaviour [41]. Future research into the strategies used by online gambling providers, and into gambling marketing strategies in general, could usefully apply this framework as a means of understanding different strategies and their likely impact on users.

## Conclusions

Overall, this study of ten popular UK online bingo sites has provided a detailed insight into features of these sites which potentially attract and retain users. Textual, graphic and content elements in the ten websites combined to attract the attention of new users and encourage them to play, to create feelings of belonging and build relationships with users, and to encourage users to step up their involvement by spending and playing more. We complemented the thematic content analysis of the bingo websites with secondary analysis of qualitative interviews with bingo players, and this has suggested that many of the features offered by online bingo websites fit well with the benefits sought by bingo players themselves. Evidence is emerging to suggest that some bingo play has the potential to escalate to problem levels, and this may be exacerbated by the immersive nature of online bingo play. Ongoing scrutiny of the content and promotion of online bingo is needed.

## Supporting Information

S1 File Protocol. Study protocol for collecting data from bingo websites.
(DOCX)

S2 File Coding frame. Coding frame for website thematic content analysis.
(DOCX)

S3 File Framework matrices. Coded data entered into framework matrices.
(DOCX)
