# Supplementary material for: The Online Bingo Boom in the UK: A Qualitative Examination of Its Appeal
Source: PLoS One. 2016 May 3;11(5):e0154763. doi: 10.1371/journal.pone.0154763 (PMC4854447; doi:10.1371/journal.pone.0154763)
Supplement: S1 File — Study protocol for collecting data from bingo websites. (DOCX) [file pone.0154763.s001.docx]

**S1 File. Study protocol for collecting data from bingo websites.**

**Website Content Protocol**

**To capture an image of the website pages (screengrab), use the NCapture web browser extension for NVivo or the Print Screen function. (Note that NCapture does not pick up all elements in a page e.g. auto-advancing images and videos.)*

| **Name of Auditor:** |  |
| --- | --- |
| **Date:** |  |
| **Time:** |  |
| **Website homepage address:** |  |

**Is this a bingo only website?**

*(Tick one box)*

|  | Yes |
| --- | --- |
|  |  |
|  | No |

**PROCEDURE**

**HOME PAGE** 🡪 Capture the Home page*

1. Which of the following content pages/sub-pages are linked to on the Home page (eg. shown as page tabs at the top of the page, or as boxes on the page)?

*(Tick all that apply/write in)*

|  | Play/bingo (play bingo now or find out about upcoming bingo games) | 🡪 Capture this page* |
| --- | --- | --- |
|  |  |  |
|  | Join now/registration (instructions on how to join) | 🡪 Capture this page* |
|  |  |  |
|  | Community (chat, forums, member news, previous winners) | 🡪 Capture this page* |
|  |  |  |
|  | Promotions (special offers) | 🡪 Capture this page* |
|  |  |  |
|  | Club (loyalty scheme) | 🡪 Capture this page* |
|  |  |  |
|  | Other games (eg. slot games) | 🡪 Capture this page* |
|  |  |  |
|  | Help page | 🡪 Capture this page* |
|  |  |  |
|  | Others (write in) | 🡪 Capture this page / these pages* |

2. Is there a Site map?

*(Tick one box)*

|  | Yes | If Yes 🡪 Capture this page* |
| --- | --- | --- |
|  |  |  |
|  | No |  |

3. Are any of the following shown on the **Home page**?

*(Tick all that apply)*

|  | Info on age restrictions (excluding logos) |
| --- | --- |
|  |  |
|  | Link to info on self-exclusion options (excluding logos) |
|  |  |
|  | Responsibility initiative logos |
|  |  |
|  | Others *(write in)*  ………………………………………………………………………………………….. |

**JOINING/REGISTRATION [go to the Joining/Register/Sign up page]**

4. Does the registration page display any age restriction logos?

*(Tick one box)*

|  | Yes |
| --- | --- |
|  |  |
|  | No |

5. What age verification procedures are used?

*(Tick all that apply)*

|  | Enter date of birth |
| --- | --- |
|  |  |
|  | Check box (I am of legal age/18) |
|  | Other *(write in)*  ………………………………………………………………………………………….. |

6. Is it possible to enter a date of birth under 18 years old (eg. 1999) and to continue with the registration process?

*(Tick one box)*

|  | Yes |
| --- | --- |
|  |  |
|  | No |

7. Does the website run any check or verification on personal contact details (address, phone number, email address) before activating the account?

*(Tick one box)*

|  | Yes |
| --- | --- |
|  |  |
|  | No |
|  | Unclear *(write in)*  ………………………………………………………………………………………….. |

8. Does the registration page include a section on depositing funds?

*(Tick one box)*

|  | Yes | Continue with Q9 |
| --- | --- | --- |
|  |  |  |
|  | No | Go to Q10 |

9. Does the registration page offer any registration bonuses if you open an account and register payment details?

*(Tick one box)*

|  | Yes |
| --- | --- |
|  |  |
|  | No |

10. Are you required to deposit funds before you can complete registration?

*(Tick one box)*

|  | Yes |
| --- | --- |
|  |  |
|  | No |

11. What payment types are accepted?

*(Tick all that apply)*

|  | Credit/Debit Card |
| --- | --- |
|  |  |
|  | Pay Pal |
|  |  |
|  | Neteller |
|  |  |
|  | Skrill (Money Bookers) |
|  |  |
|  | Clickandbuy |
|  |  |
|  | Ukash |
|  |  |
|  | Paysafecard |
|  |  |
|  | Bank Transfer |
|  |  |
|  | Wire Transfer |
|  |  |
|  | Western Union |
|  |  |
|  | Cheque/draft/P.O. |
|  |  |
|  | Click 2 Pay |
|  |  |
|  | EcoCard |
|  |  |
|  | Pre Paid Card |
|  |  |
|  | Ezi Pay |
|  |  |
|  | InstaDebit (Electronic Check) |
|  |  |
|  | EntroPay |
|  |  |
|  | Instant Banking (Deposits from bank account) |
|  |  |
|  | Other(s) *(write in)*  ……………………………………………………………………………………………………………………………… |

12. If credit card details are required, does the website run any credit check or verification before activating the account?

*(Tick one box)*

|  | Yes |
| --- | --- |
|  |  |
|  | No |
|  |  |
|  | Not known |

13. Are you required to enter a chat name which is different from your username?

*(Tick one box)*

|  | Yes |
| --- | --- |
|  |  |
|  | No |

14. Does the website have a page for welcoming new members?

*(Tick one box)*

|  | Yes |
| --- | --- |
|  |  |
|  | No |

15. Does the welcome page direct or encourage new members to play any practice/free games?

*(Tick one box)*

|  | Yes |
| --- | --- |
|  |  |
|  | No |

16. Is it possible to play any of the following without depositing funds?

*(Tick all that apply)*

|  | Bingo |
| --- | --- |
|  |  |
|  | Games |
|  |  |
|  | Casino |

17. Do any of the free play games have cash prizes?

*(Tick one box)*

|  | Yes |
| --- | --- |
|  |  |
|  | No |

18. Are there any restrictions on how many games you can play for free?

*(Tick one box)*

|  | Yes |
| --- | --- |
|  |  |
|  | No |
|  |  |
|  | Not known |

19. Are you able to post or take part in chats/lobbies?

*(Tick one box)*

|  | Yes |
| --- | --- |
|  |  |
|  | No |

**BINGO PLAY OPPORTUNITIES**

20. Does the website offer ‘play bingo now’?

*(Tick all that apply)*

|  | Yes, on the Home page |  |
| --- | --- | --- |
|  |  |  |
|  | Yes, on the Play page |  |
|  |  |  |
|  | Yes, on another page *(write in)*………………….………………………..  …………………………………………………..…………………………………..…… | 🡪 Capture this page* |
|  |  |  |
|  | No |  |

21. Is it possible to play bingo without joining/signing in?

*(Tick one box)*

|  | Yes | If Yes 🡪 Capture this page* |
| --- | --- | --- |
|  |  |  |
|  | No |  |

22. What other opportunities are provided for bingo play?

*(Tick all that apply/write in)*

|  | Schedule of forthcoming live games or jackpots |
| --- | --- |
|  |  |
|  | Information on tournaments |
|  |  |
|  | Facility to pre-buy cards if cannot make the game time |
|  |  |
|  | ‘Learn to play bingo’ instructions |
|  |  |
|  | Link to a mobile app for playing bingo ‘on the go’ |
|  |  |
|  | Others *(write in)* ……………………………………………………………………………………………………..  …………………………………………………………………………………………………………..……………………  …………………………………………………………………………………………………………..……………………  …………………………………………………………………………………………………………..…………………… |

23. Is information on prize money provided?

*(Tick one box/write in)*

|  | Yes. Write in range of prize amounts, lowest to highest:  …………………………………………………………………………………………………………..…………………… |
| --- | --- |
|  |  |
|  | No |

24. Is information on game fees provided?

*(Tick one box/write in)*

|  | Yes. Write in range of game fees, lowest to highest (excluding free games):  …………………………………………………………………………………………………………..…………………… |
| --- | --- |
|  |  |
|  | No |

25. Are free bingo cards/tickets/games available?

*(Tick one box)*

|  | Yes |
| --- | --- |
|  |  |
|  | No |

**OTHER PLAY OPPORTUNITIES**

26. Does the website offer other opportunities to play games?

*(Tick all that apply)*

|  | Yes, on the Home page |  |
| --- | --- | --- |
|  |  |  |
|  | Yes, on the Play page |  |
|  |  |  |
|  | Yes, on another page *(write in)*………………….………………………..  …………………………………………………..…………………………………..…… | 🡪 Capture this page* |
|  |  |  |
|  | No |  |

27. What play opportunities are offered?

*(Tick all that apply/write in)*

|  | Slot games |
| --- | --- |
|  |  |
|  | Instant games/scratchcards |
|  |  |
|  | Casino games (eg. roulette, blackjack) |
|  |  |
|  | Lotteries |
|  |  |
|  | Poker |
|  |  |
|  | Sport betting (eg. horse racing, football betting) |
|  |  |
|  | Other games (write in) …………………………………………………………………………………….……..  …………………………………………………………………………………………………………..……………………  …………………………………………………………………………………………………………..……………………  …………………………………………………………………………………………………………..…………………… |

28. Is it possible to play these games without joining/signing in?

*(Tick one box)*

|  | Yes | If Yes 🡪 Capture this page* |
| --- | --- | --- |
|  |  |  |
|  | No |  |

**COMMUNITY/CHAT/FORUM** 🡪 Capture this page/pages*

29. Which of the following community options can be viewed?

*(Tick all that apply/write in)*

|  | Without joining/registration | After joining/registration |
| --- | --- | --- |
| Information about games hosts |  |  |
|  |  |  |
| Information about celebrity game hosts |  |  |
|  |  |  |
| Information/profiles of other players |  |  |
|  |  |  |
| Chat room/forum |  |  |
|  |  |  |
| Blog |  |  |
|  |  |  |
| Invite a friend |  |  |
|  |  |  |
| Previous winners |  |  |
|  |  |  |
| Instant messaging facility |  |  |
|  |  |  |
| Others *(write in)……………………………………….*  *…………………………………………………………………*  *…………………………………………………………………*  *…………………………………………………………………* |  |  |

**PROMOTIONS**

30. Does the website show promotions/offers?

*(Tick all that apply/write in)*

|  | Yes, on the Home page |  |
| --- | --- | --- |
|  |  |  |
|  | Yes, on the Promotions/Offers page |  |
|  |  |  |
|  | Yes, on another page *(write in)*………………….………………………..  …………………………………………………..…………………………………..…… | 🡪 Capture this page* |
|  |  |  |
|  | No |  |

31. What types of promotions/offers are shown?

*(Tick all that apply/write in)*

|  | Big prize money |
| --- | --- |
|  |  |
|  | Free games |
|  |  |
|  | Discounted game fees |
|  |  |
|  | Bonus Cash when joining |
|  |  |
|  | Special prizes on specific days/games |
|  |  |
|  | Bonus for referring a friend |
|  |  |
|  |  |
|  |  |
|  | Others *(write in)* ……………………………………………………………………………………………..……..  …………………………………………………………………………………………………………..……………………  …………………………………………………………………………………………………………..……………………  …………………………………………………………………………………………………………..…………………… |

32. Are there any special/promotional offers for new members (eg. welcome bonuses, £XX free play)?

*(Tick one box)*

|  | Yes | If Yes 🡪 Capture this page* |
| --- | --- | --- |
|  |  |  |
|  | No |  |

**CLUB/LOYALTY SCHEME**

33. Does the website have a loyalty scheme (points are rewarded for play/website use and redeemable for further games or ££)?

*(Tick all that apply/write in)*

|  | Yes, on the Home page |  |
| --- | --- | --- |
|  |  |  |
|  | Yes, on the Promotions/Offers page |  |
|  |  |  |
|  | Yes, on another page *(write in)*………………….………………………..  …………………………………………………..…………………………………..…… | 🡪 Capture this page* |
|  |  |  |
|  | No |  |

34. Does the website have a VIP players’ club/membership scheme (eg. minimum deposit requirements)?

*(Tick all that apply/write in)*

|  | Yes, on the Home page |  |
| --- | --- | --- |
|  |  |  |
|  | Yes, on the Promotions/Offers page |  |
|  |  |  |
|  | Yes, on another page *(write in)*………………….………………………..  …………………………………………………..…………………………………..…… | 🡪 Capture this page* |
|  |  |  |
|  | No |  |

35. Are there different levels of membership?

*(Tick one box)*

|  | Yes | If Yes 🡪 Capture this page* |
| --- | --- | --- |
|  |  |  |
|  | No |  |

**SELF EXCLUSION**

36. Does the website have information about a self exclusion policy?

*(Tick all that apply/write in)*

|  | Yes, on the Home page |  |
| --- | --- | --- |
|  |  |  |
|  | Yes, on the Promotions/Offers page |  |
|  |  |  |
|  | Yes, on another page *(write in)*………………….………………………..  …………………………………………………..…………………………………..…… | 🡪 Capture this page* |
|  |  |  |
|  | No |  |

**CUSTOMER SERVICE LIVE CHAT**

37. Does the website have a Customer Service ‘Live Chat’ function?

*(Tick one box)*

|  | Yes | If Yes 🡪 Capture this page* |
| --- | --- | --- |
|  |  |  |
|  | No |  |

**OTHER PROMOTIONAL CHANNELS**

38. Does the website feature links to TV adverts?

*(Tick one box)*

|  | Yes | If Yes 🡪 Capture this page* |
| --- | --- | --- |
|  |  |  |
|  | No |  |

39. Does the website feature links to social media channels? (E.g. Facebook, Twitter, YouTube)

*(Tick one box)*

|  | Yes | If Yes 🡪 Capture this page* |
| --- | --- | --- |
|  |  |  |
|  | No |  |
