# Supplementary material for: The Online Bingo Boom in the UK: A Qualitative Examination of Its Appeal
Source: PLoS One. 2016 May 3;11(5):e0154763. doi: 10.1371/journal.pone.0154763 (PMC4854447; doi:10.1371/journal.pone.0154763)
Supplement: S2 File — Coding frame for website thematic content analysis. (DOCX) [file pone.0154763.s002.docx]

**S2 File. Coding frame for content analysis.** Design and textual elements (categories A to E), and message themes and tie-ins (categories F to J) coded for each bingo website.

|  |
| --- |
| **A. COLOUR** |
| A1. Bright |
| A2. ‘Female’ (pink, purple) |
| A3. Serious (dark blue, dark green) |
| A4. Other |
|  |
| **B. IMAGERY** |
| B1. Cartoon-like/clip-art |
| B2. Animals |
| B3. Use of photos of real people |
| B4. Images of wealth/winning (trophies, gold) |
| B5. Other |
|  |
| **C. TYPOGRAPHIC** |
| C1. Use of sans serif |
| C2. Cartoon-like (eg. large caps with borders) |
| C3. Use of lower case ie. no initial caps |
| C4. Use of exclamation marks |
| C5. Use of contrasting fonts on same page |
| C6. Other |
|  |
| **D. OVERALL DESIGN** |
| D1. Cluttered |
| D2. Clean |
| D3. Text heavy |
| D4. Text light/image heavy |
| D5. Use of call-outs (banners, flashes etc) |
| D6. Rolling banners/changing images |
| D7. Other |
|  |
| **E. TEXTUAL** |
| E1. Imperatives/injunctives (‘play now’, ‘join today’) |
| E2. Catchy word play (‘Bingo Lingo’, ‘Fab Friday’) |
| E3. Demotic/colloquial/incorrect (eg. ‘me and my pals was…’) |
| E5. Use of first and second person (‘**I/we** hope **you** will….’) |
| E6. Use of third person (‘Players are reminded that…’) |
| E7. ‘Legal’ or advisory language |
| E8. Other |
|  |
| **F. MESSAGE THEMES: Recruitment, relationships** |
| F1. Join in/community/club |
| F2. Friendship/people just like you/friendly |
| F3. Initiation/mastery of new rules and practices |
| F4. Socialising, chat |
| F5. Excitement |
| F6. Fun, lightheartedness |
| F7. Reassurance |
| F8. References to club’s/community’s own rules, currency and language (eg. ‘CPs’) |
| F9. Other |
|  |
| **G. MESSAGE THEMES: Play and winning** |
| G1. ‘Free’ play money |
| G2. Luck/chance |
| G3. Caution/risk |
| G4. Winning = normal, achievable |
| G5. Winning = treat, luxury |
| G6. Winning = financial security |
| G7. Winning = transform life/fairytale/dream come true |
| G8. Low stakes eg. play with 1p |
| G9. Incitement to play more to win more |
| G10 Consolation play/invitation to win after losing |
| G11 Other |
|  |
| **H. MESSAGE THEMES: Loyalty** |
| H1. Gain points/credits in general |
| H2. Gain points/credits in return for recruiting new players |
| H3. Better/deeper experience for more loyal players |
| H4. Exclusivity |
| H5. Other |
|  |
| **I. MESSAGE THEMES: Game features** |
| I1 References to playing frequently |
| I2 References to playing every day |
| I3 References to playing round the clock including at night |
| I4 Variety of games |
| I5 Games for individual/you |
| I6 Imminence/urgency eg. countdown |
| I7 Purchase in advance play later |
| I8 Other |
|  |
| **J. TIE-INS** |
| J1 News and current events eg. Royal wedding |
| J2 TV shows and films |
| J3 Characters (comics, films, games) |
| J4 Sport (do we need to clarify eg. not in a betting context?) |
| J5 Other |
|  |
| **K. Other** |
|  |
