# Supplementary material for: The Online Bingo Boom in the UK: A Qualitative Examination of Its Appeal
Source: PLoS One. 2016 May 3;11(5):e0154763. doi: 10.1371/journal.pone.0154763 (PMC4854447; doi:10.1371/journal.pone.0154763)
Supplement: S3 File — Coded data entered into framework matrices. (DOCX) [file pone.0154763.s003.docx]

**Stead et al Online bingo study: coding matrices**

**1. Design and Textual elements**

|  | **Company1** | **Company2** | **Company3** | **Company4** | **Company5** | **Company6** | **Company7** | **Company8** | **Company9** | **Company10** |
| --- | --- | --- | --- | --- | --- | --- | --- | --- | --- | --- |
| **A. COLOUR** |  |  |  |  |  |  |  |  |  |  |
| A1. Bright | All pages - *but not clashing, red and yellow* | All pages | All pages | All pages - *but not clashing, blue and yellow* |  | Home-Promotions-Bingo  Home-Community-ChatGames  Home-login/play now | All pages |  | All pages | Bingo games |
| A2. ‘Female’ (pink, purple) |  | All pages |  |  | All pages |  |  |  | All pages |  |
| A3. Serious (dark blue, dark green) |  |  |  |  |  |  |  | All pages |  | All pages |
| A4. Other |  |  |  |  |  | Most pages |  |  |  | All pages – *‘fresh’ colours associated with healthy/’lite’ advertising, also M&S* |
|  |  |  |  |  |  |  |  |  |  |  |
| **B. IMAGERY** |  |  |  |  |  |  |  |  |  |  |
| B1. Cartoon-like/clip-art | Games – bingo slots  Homepage – promotions  Community – chat hosts | Homepage  Homepage – Register  Games chatgames  Homepage – Community  Community – Cheeky and You  Community – Winners  Homepage – FAQs  Promotions – Loyalty points  Games – pre-buy bingo  Responsibility  Games – guaranteed jackpots  Promotions – slots & casino  All Stars VIP club p1-4  Homepage - Help | All pages | Games bingo  Homepage help  Homepage – Promotions – Community  Promotions –Monthly Promotions | Home-Bingo | Home-Community-ChatGames |  | Homepage  Play now p1-2  Community  Promotions p1-2 | Home, Bingo, Slots, Promotions, Scratchcards, Star Rewards | Bingo games  Chatroom moderators |
| B2. Animals |  |  |  | Games bingo – *Karate Pig*  Games more – *Karate Pig*  Games progressive jackpots - *Karate Pig, cartoon zebra*  Games scratchards – *Karate Pig*  Games slots – *Karate Pig, cartoon zebra*  Games tablegames – *Karate Pig*  Homepage help – *Karate Pig*  Games promotions - *Karate Pig, cartoon giraffe* | All pages – foxy | Home p2 |  |  | Home |  |
| B3. Use of photos of real people | All – ‘*need help? click here’ includes photo of woman w phone headset*  Homepage – *still from TV ad (woman dancing); woman cheering beside list of winners*  Homepage Promotions 1 and 2 – *still from TV ad*  Games – bingo slots – *still from TV ad*  Community player testimonials | Homepage – Community  Community – Winners  Games – guaranteed jackpots  All Stars VIP club p4 | Home- winners  Blog | Homepage – *4 recent winners’ photos rotate* | Community-ChatHosts | Home-Promotions-VIP |  | Homepage | Mobile | Home  Promotions  Community 2 Winners  Community Previous winners  Other games  Mobile app |
| B4. Images of wealth/winning (trophies, gold) | Homepage – Promotions | Homepage  Homepage – Community  Community – Winners  All Stars VIP club p1-4 - *diamonds* | Home-blog  Promotions | Homepage – ‘*gold bar’ font for ‘THE BIG £10K’*  Homepage promotions – *moneybag, trophy, jackpot*  Homepage – Promotions – Community – *piggy bank*  Promotions –Monthly Promotions – *pot of gold* | Home-Bingo | Home-Promotions-Bingo *– image of Vegas*  Home-Promotions-VIP | Penny Bingo – images of pennies.  Big Games – images of gold | Homepage – *cheering crowd silhouette*  Promotions p1 – *another cheering/dancing crowd silhouette* | Home, Bingo | Home  Promotions  Other games |
| B5. Other | All – funfair imagery/theme *(rollercoaster, big wheel, circus tent*) | All Stars VIP club p1-4 - *photo of prize (Samsung tablet)* | All pages – *sun;*  Games – *images of other gambling* |  |  | Home p1 – gold dust swoosh to front room |  |  | Promotions – images of biscuits (tea break) | Home – *images of outdoors (picnics, walks etc)* |
|  |  |  |  |  |  |  |  |  |  |  |
| **C. TYPOGRAPHIC** |  |  |  |  |  |  |  |  |  |  |
| C1. Use of sans serif | All pages | Homepage  Homepage – Register  Games chatgames  Games chatgames  Community – Cheeky and You  Community – Winners  Homepage – FAQs  Promotions – Loyalty points  Games – pre-buy bingo  Responsibility  Games – guaranteed jackpots  Promotions – slots & casino  All Stars VIP club p1-4  Homepage - Help | All pages | All pages | All pages | All pages | Penny Bingo | All pages | All pages | Home  Bingo games |
| C2. Cartoon-like (eg. large caps with borders) | Homepage – Promotions  Promotions 1  Promotions 2  Promotions 3 | Homepage  Homepage – Register  Games chatgames  Games chatgames  Community – Winners  Homepage – FAQs  Promotions – Loyalty points  Games – pre-buy bingo  Responsibility  Games – guaranteed jackpots  Promotions – slots & casino  All Stars VIP club p1-4  Homepage - Help | All pages | Homepage  Promotions –Monthly Promotions – Games Bingo  Homepage promotions | All/most pages | Home-Promotions-Bingo  Home-Promotions-VIP | Big Games | Homepage  Promotions | Bingo, Slots, Promotions, Other games |  |
| C3. Use of lower case ie. no initial caps |  | Homepage – *usernames*  Games chatgames  Community – Winners  Community – Winners  Homepage – FAQs  Promotions – Loyalty points  Games – pre-buy bingo  Responsibility  Games – guaranteed jackpots  Promotions – slots & casino | Home-join |  |  |  |  | Play now p1-2  Help pages 1-3  Joining promotion  *“mirrorbingo” in these pages but “Mirror Bingo” elsewhere* |  | Home  Bingo games |
| C4. Use of exclamation marks | Homepage – Other Games  Homepage – Promotions  Homepage – Schedule  Promotions 1  Promotions 2  Promotions 3  Games – 90 ball bingo  Games – bingo slots  Community – Chat Games  Community – Chat hosts | Homepage  Homepage – Register  Games chatgames  Games chatgames  Community – Cheeky and You  Community – Winners  Homepage – FAQs  Promotions – Loyalty points  Games – pre-buy bingo  Games – guaranteed jackpots  Promotions – slots & casino  All Stars VIP club p1-4 |  | Games bingo  Games progressive jackpots  Games slots | All/most pages | Home p1  Home-Promotions-VIP  Home-Community-Refer-a-friend | Promotions | Homepage  Play now p1-2  Community  Promotions p1-2  Winners  Testimonials  Loyalty club |  | Bingo games  Blog |
| C5. Use of contrasting fonts on same page |  | Games chatgames  Community – Cheeky and You  Community – Winners  Homepage – FAQs  Promotions – Loyalty points  Games – pre-buy bingo  Promotions – slots & casino  All Stars VIP club p1-4  Homepage - Help | Games-slots | Homepage | All/most pages | Home-Promotions-Bingo  Home-Community-ChatGames |  | Homepage  Promotions | Bingo, Slots, Other games |  |
| C6. Other |  |  | All pages – *‘play now’ button resembles Lego logo* |  |  |  |  |  |  |  |
|  |  |  |  |  |  |  |  |  |  |  |
| **D. OVERALL DESIGN** |  |  |  |  |  |  |  |  |  |  |
| D1. Cluttered |  | Homepage *– grid layout*  All Stars VIP club p2-3 – *previous promotions* | Home | Homepage – *although in a grid layout, each ‘cell’ contrasts with its neighbours by colour, font, content* | Home  Mobile | Home-login/play now |  |  |  |  |
| D2. Clean |  | Homepage – Register  Games chatgames  Homepage – FAQs  Promotions – Loyalty points  Games – pre-buy bingo  Responsibility  Games – guaranteed jackpots  Promotions – slots & casino  Homepage - Help |  |  |  | Home p1 | All pages | All pages | All pages | Home |
| D3. Text heavy | Homepage – about us  Homepage – Banking  Homepage – Help FAQs  Homepage – Help  Homepage – Join now  Games – 90 ball bingo  Community – chat games  Community – chat lingo  Loyalty – BBZ 2 | Homepage – Register  Games chatgames  Community – Winners  Homepage – FAQs  Responsibility  Games – guaranteed jackpots |  | Homepage – help  Homepage – promotions  Loyalty  Refer a friend  T&Cs  Bonus money 1  Bonus money 2  Responsible Gaming |  | Home-Responsible Gaming | Contact us, Mini games | Join now  Help pages 1-3  Winners  Testimonials  Joining promotion  Loyalty club  Self exclusion  Self limit |  | Help page 1  Self exclude  Prices  Prizes |
| D4. Text light/image heavy |  |  |  |  |  |  |  |  | Mobile |  |
| D5. Use of call-outs (banners, flashes etc) | Homepage - schedule | Homepage – Register |  | Homepage – *text across recent winners’ photos* |  | Home-Promotions-VIP |  |  |  |  |
| D6. Rolling banners/changing images | Homepage |  |  | Homepage – *4 recent winners’ photos rotate; rolling list of top rated games* |  |  |  |  | Home, Promotions |  |
| D7. Other |  |  | Home-join |  |  |  |  | All – *Mirror newspaper logo v small* |  | Home *– use of white space* |
|  |  |  |  |  |  |  |  |  |  |  |
| **E. TEXTUAL** |  |  |  |  |  |  |  |  |  |  |
| E1. Imperatives/injunctives (‘play now’, ‘join today’) | All – *‘Play Bingo’ and ‘Join now’ buttons* | Homepage  Homepage – Register  Games chatgames  Promotions – Loyalty points  Games – pre-buy bingo  Promotions – slots & casino  Promotions – slots & casino  All Stars VIP club p1-4  Homepage – Help – *‘Get in touch’* | All pages | Homepage  Games bingo  Games more  Games progressive jackpots  Games scratchcards  Games slots  Games tablegames  Homepage help  Homepage games  Homepage – Promotions – Community | Home  Promotions  Mobile | Home p1 and all pages | Penny Bingo – ‘Register now’, ‘Make sure you grab your penny tickets today’. | Homepage – *‘grab a share’*  Promotions p1 – *‘join now!’*  Joining promotion | Promotions – *‘This is your moment’, ‘Today’s the day’.* | Home |
| E2. Catchy word play (‘Bingo Lingo’, ‘Fab Friday’) | Community – chat games – *double trouble*  Games – bingo slots – *Jungle Jim bingo, Cashanova, Mega Moolah*  Community – chat lingo  Bingo schedule – *‘fabby fun bingo games’, ‘spreading the lurve’* | Homepage – *‘Play for free. Win for real!’*  Homepage – Register  Games chatgames  Community – Winners  Promotions – slots & casino  All Stars VIP club p1-4  Homepage - Help | Home- winners  Games-slots  Promotions | Homepage – ‘*top treats’, ‘ravishing rewards’*  Games more – *‘bingo bonanza’*  Games progressive jackpots – *‘major millions’, ‘mega moolah’*  Homepage promotions – ‘*consolation station’; ‘cool Consolation Prize Free Spins’; ‘fantastic freebie’*  Homepage – Promotions – Community – *Eggs Up*  Promotions –Monthly Promotions *– ‘twice as nice!’* | Home  Promotions | Home p1 bingo buzz, hot slots  Home-Promotions-Bingo  Home-Promotions-VIP  Home-Community-ChatGames | Penny Bingo – ‘Cash climber’.  Big Games – ‘Bingo Linx’.  Bingo schedule – ‘Pit stop’, ‘joker jackpot’ etc.  Top games – ‘Cash blox, clover rollover’. | Community – *‘bingo lingo’* | Slots – ‘*Make Money’, ‘Clover Rollover* |  |
| E3. Demotic/colloquial/incorrect (eg. ‘me and my pals was…’) | Bingo schedule – *‘spreading the lurve’*  Bingo testimonials |  | Home- winners  Blog  Jackpots | About us – *use of & and ‘and’ in same sentence: ‘is owned & operated … industry and they all love…’*  Promotions –Monthly Promotions – *prize* ‘*YSL Fragerance [sic]’* | Home-Family-RFLMuM  Community-ChatHosts  Community-SuperLeague |  |  |  |  | Chatroom moderators |
| E5. Use of first and second person (‘**I/we** hope **you** will….’) | All pages | Homepage  Homepage – Register  Games chatgames  Homepage – Community  Community – Cheeky and You  Homepage – FAQs  Promotions – Loyalty points  Games – pre-buy bingo  Responsibility  Games – guaranteed jackpots  Promotions – slots & casino  All Stars VIP club p1-4  Homepage - Help | Home  Blog  Help | All pages | Home  Promotions-Loyaltypoints  Home-Bingo  Community-SuperLeague |  | Contact us- | All pages (*even the Help/Legal pages)* | Home – *‘Me time’.*  Promotions – *‘This is your moment’*.  Star Rewards – *‘your star level’, ‘your bonus’.* | About us – *‘We’re always really happy to hear from you’* |
| E6. Use of third person (‘Players are reminded that…’) | Loyalty – BBZ 2 ‘*Most players are honest and trustworthy, however…’* | Homepage – FAQs |  | T&Cs  Refer a friend  Bonus money 1  Bonus money 2 |  | Home-Promotions-Loyalty |  |  |  |  |
| E7. ‘Legal’ or advisory language | Homepage – Banking  Loyalty – BBZ 2 | Homepage – FAQs  Responsibility | Home- winners  Home-Responsible | T&Cs  Bonus money 2  Responsible Gaming | Home-Responsible | Home-Responsible Gaming | Super free bingo |  |  | Help page 1 |
| E8. Other |  |  | Home – *contrast ‘deposit x, play with y’* |  | Home-Bingo – *variety of descriptions for play and winning*  Community-Followme – *LOL;*  Community-Followme  *personalised talking mascot (most pages)*  Community-SuperLeague  *personalised talking mascot (most pages* |  | Promotions – ‘Cash climber – reach the top of the escalator and win £20,000’ Encourages a deeper experience. |  |  | Home *“…great promotions every week; there is always something to suit everyone”. Interesting use of semi-colon.*  About us – *‘we’ll keep trying to make it better’. Assumed modesty, unlike eg. Foxy, Cheeky. Contributes to overall impression of moderation, safety, politeness* |
|  |  |  |  |  |  |  |  |  |  |  |
|  |  |  |  |  |  |  |  |  |  |  |

**2. Message themes and tie-ins**

|  | **Company1** | **Company2** | **Company3** | **Company4** | **Company5** | **Company6** | **Company7**  **(Old)** | **Company7 (New)** | **Company8** | **Company9 (Old)** | **Company9 (New)** | **Company10** |
| --- | --- | --- | --- | --- | --- | --- | --- | --- | --- | --- | --- | --- |
| **F. MESSAGE THEMES: Recruitment, relationships** |  |  |  |  |  |  |  |  |  |  |  |  |
| F1. Join in/community/club | Community – Chat games | Homepage – Register  Games chatgames  Homepage – Community  Community – Cheeky and You  Community – Winners  All Stars VIP club p1-4 | Blog | Homepage  Homepage promotions | Home  Community-Magazine | Home p2 |  |  | Play now p2 – *‘Chat games and Tourneys’ [tournaments]* | News and Community – ‘Hello roomies’. | Promotions – *‘You make the tea, we’ll bring the fun’*, *‘social perks’*. | Bingo games *– ‘join in the fun’*  Chatroom moderators – *friends, lovely and friendly* |
| F2. Friendship/people just like you/friendly | Homepage – play now registration - *‘the internet’s friendliest online bingo hall’*  Community – chat hosts – *profiles*  Homepage - Bingo schedule – ‘*Friendship Lounge’*  Homepage community – *‘opportunity to meet life-long friends’* | Games chatgames *– game called ‘Be My Friend’*  Homepage – Community  Community – Winners  All Stars VIP club p1-4 | Home- winners  Blog | Homepage – *‘get to know other roomies and our Cheery Chat Hosts’; ‘meet your friendly bingo community’*  Homepage help  Games Bingo – *90 ball bingo* | Home – ‘foxy family’  Community-ChatHosts  Community-Winners |  |  | On the house rewards – ‘roomies’. | Testimonials – ‘*we have a good laugh’, ‘everyone is really friendly’* | News and Community – ‘You have lovely friends who have recommended you to win’. |  | About us – *stresses that customer service reps ‘mostly based in Sunderland actually!’.*  Chatroom moderators – *‘a lil family online’* |
| F3. Initiation/mastery of new rules and practices | Games – 90 ball bingo – *full page instructions [all text]* | Games – pre-buy bingo  Homepage - Help | Help  Promotions – *‘first time depositor’* | Homepage – *‘become a fully-fledged … roomie with your very own nickname’*  Homepage help | Community-ChatHosts  Help | Home p3 |  |  | Community – *Bingo Lingo*  and *Bingo Calls, ‘be a real bingo pro’* |  |  | Community 1 *– Binglish translator*  Chatroom moderators |
| F4. Socialising, chat | Community – chat hosts – *profiles*  Community – chat games  Bingo schedule – *‘roomie chums’, ‘fellow roomies,* | Games chatgames  Homepage – Community  Community – Winners  Promotions – Loyalty points | Blog | Homepage – *‘where you can chat and play’*  Games bingo – *90 ball bingo* | Home  Community-ChatHosts  Community-Magazine – ‘goss’ |  | Bingo Lingo chat – ‘Bingo is a social thing, so get yourself involved ppl!’. | Bingo Lingo chat – ‘Bingo is a social thing, so get yourself involved ppl!’. | Testimonials – *‘a great way of socializing, as I can’t go out’* |  | Promotions – *‘social perks’*. | Home – *‘friendly chat rooms’*  Chatroom moderators |
| *F5. Excitement* |  | Community – Winners  Promotions – slots & casino  All Stars VIP club p1-4 |  |  |  |  |  | Home – ‘Go on an adventure to Wonderland’.  Our Bingo Games – ’80 ball bingo gives you the added excitement’. |  |  |  |  |
| *F6. Fun, lightheartedness* |  | Homepage – Community  Community – Winners  Promotions – slots & casino  Homepage - Help |  |  |  | Home-Promotions-VIP |  | Getting started – ‘so go have some fun and good luck!’ |  | Home – ‘For fun’s sake, play bingo’. | Promotions – *‘spreading bingo cheer’*. |  |
| F7. Reassurance | Homepage – *‘rest assured that our award-winning bingo support team will be there to assist you’*  Homepage - Help | Homepage – Register *– no obligation card details*  Homepage – Community - *‘feel a part of the gang’, ‘You’ll love…’*  Homepage – FAQs  Games – pre-buy bingo  Responsibility  Games – guaranteed jackpots – *‘never miss out…’*  Homepage - Help | Help  Refer a Friend | Homepage promotions – *refer a friend: ‘It’s a win-win situation, don’t miss out!’*  About us – *‘super-easy to use’; servers ‘can only be accessed by our highly-qualified, super-trustworthy, authorized personnel’*  Homepage – Promotions – Community *– ‘so don’t be shy’* | Community-ChatHosts  Help | Home-Promotions-VIP | Our bingo games – ‘Sound complicated? The auto-dab feature at Ladbrokes Bingo means you won’t miss out on a prize!’  Bingo Lingo chat | Our bingo games – ‘Sound complicated? The auto-dab feature at Ladbrokes Bingo means you won’t miss out on a prize!’  Getting started – ‘It’s easy to get involved’  Bingo Lingo chat | Help page p1 – ‘*registering … is simple’; ‘do I spend ages downloading software? absolutely not!’* |  |  | About us – *several references to ‘safe’*  Community 1 *– code of conduct emphasises courteous and ethical behaviour*  ?Chatroom moderators – *all named after confectionary* |
| F8. References to club’s/community’s own rules, currency and language (eg. ‘CPs’) | Loyalty – BBz *– Bingo Bonus Money*  Loyalty – BBz2  Community – Chat Lingo | Games chatgames  Homepage – Community  Community – Cheeky and You – *‘you my loyal cheekies!’*  Community – Winners  Homepage – FAQs  Promotions – Loyalty points  Responsibility  All Stars VIP club p1-4 |  | Homepage – *winner photos ‘WTG!’ [way to go!]; PJPs*  Homepage promotions  Loyalty  Bonus Money 1  Bonus Money 2  Homepage – Promotions – Community – *BBz*  Promotions –Monthly Promotions - *SJP* | Community-ChatHosts  Home-Bingo *– ‘1TG’ etc*  Community-SuperLeague | Home-Promotions-Loyalty  Home-Community-Refer-a-friend | Big games – ‘1&2TG’  Bingo Lingo chat  £50 bonus bundle – ‘Laddies Rewards’. | Big games – ‘1&2TG’  Bingo Lingo chat  News – ‘WWTBAM winner!’ | Community  Loyalty Club – *‘just ask our chat hosts for details’* | News and Community – ‘here are your free BBs’. |  | Community 1 *– chat rules* |
| F9. Other |  |  | Home – *‘Doting Dads’, Father’s Day*  Blog – *relationship with logo as person; togetherness*  Refer a Friend *– different email providers prompt* |  | Home *– our own tV and radio shows, magazine; welcome for new players*  Home-Family-RFLMuM – *‘mum of year’, real life stories* | Home-Promotions-VIP *– your every need* | Super free bingo – ‘You would be mad to miss out’. |  |  |  |  | Contact us – *chat hosts’ availability*  Help page 1 – *chat hosts’ availability* |
|  |  |  |  |  |  |  |  |  |  |  |  |  |
| **G. MESSAGE THEMES: Play and winning** |  |  |  |  |  |  |  |  |  |  |  |  |
| G1. ‘Free’ play money | Promotions 1  Promotion 2 | All pages – *logo strapline ‘Play for free. Win for real.’*  Community – Winners *‘Friday free for all … clue’s in the name’*  All Stars VIP club p1 – *Free bingo cards* | Promotions and many other pages | Homepage – *‘£15 free for all new players’; ‘Cash Castle FREE BINGO EVERYDAY’*  Homepage promotions – *Free bingo games*  Promotions – New player bonus  Homepage – Promotions – Community  Promotions –Monthly Promotions *– Free Bingo* | Mobile - *£20 free play* | Home p1  Home-Promotions-Bingo  Home-login/play now | Promotions  £50 Bonus bundle | Home – ‘Free Bingo 24 hours a day!’, ‘Spend £10 play with £40’.  Promotions – ‘Super free bingo’.  News – ‘top 5 free games’. | Promotions p1 – ‘*bonus cash when you join’*  Joining promotion | Home – ‘get your £20 freeplay’.  Promotions – ‘win free bingo for a year’. | Home, Promotions – *‘Free Bingo’*. | Bingo games |
| G2. Luck/chance | Games – 90 Ball Bingo | Games chatgames  All pages – *callout strapline ‘TOP PROMO! … more chances to win.’*  Responsibility  Promotions – slots & casino | Blog – *lucky numbers* | Homepage – *‘try your luck’*  Games Bingo – ‘*good luck to all’*  Promotions –Monthly Promotions – *‘this jackpot rises and falls at random’* |  |  | Mini Games – ‘I must admit it was a bit of a fluke…my luck was in’. | Bingo Lingo chat – ‘gl = good luck’. | Play now p1  Joining promotion – ‘*the chance to win’* | News and Community – ‘here are the lucky winners’.  Promotions – ‘two lucky winners will win’. |  |  |
| G3. Caution/risk |  | Homepage – Register |  |  |  |  |  |  |  |  |  |  |
| G4. Winning = normal, achievable | Homepage – other games - *Poker game is ‘great fun, simple to use and rewarding’* | Homepage – *‘Win for Real!’*  All pages – *logo strapline ‘Play for free. Win for real.’*  All pages – *callout strapline ‘TOP PROMO! More Jackpots More Winners!’*  Community – Winners  Games – pre-buy bingo  Games – guaranteed jackpots | Home-join – *updating list of winners*  Home-winners  Games | Homepage – *recent winners* | Community-Winners |  | Penny Bingo – ’10,000 winners a week’ – previous winners. | Home -’10,000 winners a week’ – previous winners.  ‘Win cash and prizes every day!’  Promotions – ‘Win cash and prizes every day!’, ‘Risk free Bingo’. | Winners - *‘We love it when our players win big and have fun – after all that’s what bingo’s all about’*  Winners – ‘*fantastic to win so soon after joining’*  Testimonials (same quote) | Promotions – ‘clean up with a share of £30,000’. | Promotions – *’15,000 to be won every night’*. | Home – *‘won the JP’ during a break at work*  Community 2 Winners – *long list of winners emphasises possibility* |
| G5. Winning = treat, luxury | Games – bingo slots *‘sumptuous payouts’, ‘you’ll be rolling round in money’* |  |  | Games slots – *‘epic rewards!’; ‘feel the thrill of massive rewards’* | Community-Winners |  |  |  |  | Promotions – ‘Paddy’s mile high club’. |  | Community 2 Winners – *testimonials*  Other games |
| G6. Winning = financial security |  |  |  |  | Community-Winners |  |  |  |  |  |  | Community 2 Winners - testimonials |
| G7. Winning = transform life/fairytale/dream come true | Community – chat hosts – *profiles, all answer ‘One day when I win big:…’*  Games – bingo slots – *‘capable of life changing wins in just one lucky spin’; ‘could change your life’* | Homepage |  | About us – *‘where all your bingo wishes come true!’* |  |  |  | On the House Rewards – travel from ‘the garden’ to ‘the penthouse’. |  |  | Promotions – *‘turn 50p into £8,000’* | Community 2 Winners - *testimonials* |
| G8. Low stakes eg. play with 1p | Homepage – Bingo Schedule – ‘*tickets from just 5p each!’* | Homepage – ‘*for just 1p’* |  | Homepage – ‘*tickets from just 1p’* | Promotions – *1p and 2p games* | Home-login/play now | Big games – 10p per ticket  Bingo Schedule – 5p, 2p  Penny Bingo – 1p |  |  |  | Bingo – price on display | Home – *games from 2p*  Prices |
| G9. Incitement to play more to win more | Homepage – promotions – *‘25% re-deposit bonus’* | Games – pre-buy bingo *‘Occasionally, we have special offers’* |  | Homepage promotions – *Weekend Slot – win for being player to wager the most; Bingo League, gain points for taking part in ‘league games’, weekly prizes for those with most points.*  Promotions –Monthly Promotions – *weekend slot (as above)* Loyalty *– loyalty club tiers (‘earn the required amount of maintenance Loyalty points’)*  Promotions – New player bonus – *redeposit bonus*  Promotions –Monthly Promotions – *must play for 1.5 hrs to be eligible for prizes* |  |  |  | On the House Rewards – ‘there are now 5 tiers that roomies can work their way through’. | Promotions p2 – *‘Sunday: The More You Play Tournament!’* |  |  |  |
| G10 Consolation play/invitation to win after losing |  |  |  | Homepage promotions – ‘*consolation station’*  Promotions –Monthly Promotions – ‘*cool Consolation Prize Free Spins’.* |  |  |  |  |  |  |  |  |
| G11 Other |  | Promotions – slots & casino – ‘*Cheat*[??] *on bingo with Cheeky’s fantastic range of slot and casino games!’* | Home – ‘free to enter jackpot game, world record for free bingo’  Home-join – *deposit bonus calculator [also on other pages]*  Refer a Friend *– latest winner large sum*  Refer a Friend *– “earning” some serious cash* | Homepage 2 – *one winner’s photo used $$*  Promotions – New player bonus - *£ and $ in same text [*corrected to ££ in newer website grabs*]*  Promotions – New player bonus – *complex redeposit bonus* |  | Home-Promotions-Bingo *– ipad prize, Vegas vacation* |  |  |  |  |  | Home – *‘spend the jackpot on my beautiful girls’ – family juxtaposed with winning*  Home *– use of testimonials from winners* |
|  |  |  |  |  |  |  |  |  |  |  |  |  |
| **H. MESSAGE THEMES: Loyalty** |  |  |  |  |  |  |  |  |  |  |  |  |
| H1. Gain points/credits in general | Loyalty – BBz *– Bingo Bonus Money*  Loyalty – BBz2 | Games chatgames – *extra CPs*  Homepage – Community  Community – Winners | Diamond Club | Loyalty  Promotions –Monthly Promotions – *Double loyalty points* | Promotions-Loyaltypoints | Home-Promotions-Loyalty | On the house rewards | Home  On the House Rewards | Play now p2  Winners – ‘*we give away loads of bonus Bingo Points’*  Promotions p2 – ‘*reward you every time that you come on to the site’*  Loyalty club | Star rewards | Star Rewards – *‘As you play, you can earn star points’.* | Community invite a friend |
| H2. Gain points/credits in return for recruiting new players | Homepage – promotions | Promotions – Loyalty points | Promotions  Refer a Friend *– nb. Has own page* | Homepage promotions – *refer a friend* | Promotions-Loyaltypoints | Home-Promotions-Loyalty  Home-Community-Refer-a-friend |  |  | Promotions p2 – *‘every friend you refer’* | News and Community – ‘Recommend a friend winners’. |  | Community invite a friend |
| H3. Better/deeper experience for more loyal players |  | All Stars VIP club p1-4 | Diamond Club | Loyalty | Home  Promotions-Loyaltypoints  EliteClub | Home-Promotions-Loyalty | On the house rewards  £50 bonus bundle | On the House Rewards – ‘Each level enjoying the benefits of Free Bingo, Bonus Bundles, Prize Draws and more’. |  |  | Star Rewards |  |
| H4. Exclusivity |  | All Stars VIP club p1-4 *– confined to 250 players* | Diamond Club | Homepage – *one room for ‘depositors only’*  Loyalty – *loyalty club tiers (Blue, Silver, Gold, Platinum) – Diamond and Privé (complex structure)* | EliteClub | Home-Promotions-Loyalty  Home-Promotions-VIP |  | On the House Rewards |  |  |  |  |
| H5. Other |  |  | Diamond Club – ‘girl’s best friend’ | Loyalty – *free loyalty points for new players* |  | Home-Community-Refer-a-friend – *bear toy* | Promotions – bonus points for new players |  |  |  |  |  |
|  |  |  |  |  |  |  |  |  |  |  |  |  |
| **I. MESSAGE THEMES: Game features** |  |  |  |  |  |  |  |  |  |  |  |  |
| I1 References to playing frequently | Homepage – other games – *‘In between your games of bingo you can try out our [list of games] … and can keep winning while you wait for your next Bingo game to start’* |  | Jackpots | Loyalty  Promotions –Monthly Promotions |  | Home-Promotions-VIP | Penny Bingo – ‘Play bingo on the go’. | Promotions – ‘Play bingo on the go on your smartphone or tablet’. | Promotions p2 – *‘Sunday: The More You Play Tournament!’* Loyalty club –*‘keep checking your account’* |  |  |  |
| I2 References to playing every day |  | Homepage  Homepage – Register  Games – pre-buy bingo *– 7 days listed* |  | Homepage promotions – *Weekend Slot promotions*  Promotions –Monthly Promotions | Home | Home p2  Home-Promotions-Bingo | Bingo schedule – days are listed  Penny Bingo  Super Free Bingo | Bingo schedule – days are listed | Loyalty club – *‘chat games which run every few minutes EVERY day!’* | Promotions – days are listed. | Promotions – *Specific days for certain games* | Promotions – *calendar of future games (but not as detailed or frequent as on other sites)* |
| I3 References to playing round the clock including at night | Homepage - schedule – ‘*open 24 hours’; 4 other lounges/rooms open at different times eg. 5pm-5am, noon-midnight; 6pm- midnight; 3pm-midnight* | Homepage – *until midnight*  Games – guaranteed jackpots | Home – *‘every three minutes’*  Promotions – *24 hrs a day; every 30 mins* |  | Promotions  Promotions-Loyaltypoints  EliteClub | Home-login/play now | Big Games – timetable  Bingo schedule – timetabled up to every 15 seconds  Penny Bingo  Super Free Bingo | Promotions – ‘free bingo 24 hours a day’. | Promotions p1 – *‘only on Sunday nights’* | All pages ’24 hour support’. | Promotions – *‘Rise and Shine’, ‘Take back the night’*. | Promotions – *‘breakfast bingo’* |
| I4 Variety of games | Games – bingo slots – *‘all the bingo slots you’ll ever need’*  Community chat games – *‘we have loads of different chat games’* | Promotions – slots & casino – *‘a fantastic range’* |  | Homepage – *‘Our unbeatable selection’*  Promotions –Monthly Promotions | Home-Bingo |  | Our bingo games – ‘If you thought there was only one way to play bingo the you’re in for a treat’  Mini games | Our bingo games – ‘If you thought there was only one way to play bingo the you’re in for a treat’  Bingo slots | Play now p2 – ‘*not just bingo here*’ | Promotions | Promotions – ‘cheap ticket Tuesday’. | Bingo games *– ‘cater for everyone’s budget’* |
| I5 Games for individual/you |  |  |  |  |  | Home-Promotions-Bingo – *find your promotion*  Home-Community-ChatGames – *your chat game* | Our Bingo Games | Our Bingo Games |  |  |  | Home – *something to suit everyone*  Bingo games *– ‘which will be your favourite?’* |
| I6 Imminence/urgency eg. countdown | Homepage – play now registration – *‘you’re only 2 minutes and one simple forma away from joining and starting the bingo fun’*  Homepage – Help FAQs – *‘you’ll be able to get down to some serious bingo pretty quickly’* | Homepage  Games chatgames – ‘*so what are you waiting for?’* | Home – *‘tonight at 9pm’, ‘faster sign up just 20 seconds’*  Home-join – *jackpot barometers* |  |  | Home-Promotions-Bingo – *200k weekend* | Big Games – ‘don’t miss out, buy your tickets today.  Mini games – ‘what are you waiting for? Play today!’ | Big Games – ‘don’t miss out, buy your tickets today.  Bingo schedule – countdown to games visible. |  |  | Bingo – clock counting down |  |
| I7 Purchase in advance play later | Homepage – promotions – *Weekly pre-buys, Monthly pre-buys* | Games – pre-buy bingo | Home – *‘pre-buy’* |  | Home-Bingo  Community-SuperLeague |  |  | Bingo schedule | Play now p1 – ‘*recently launched pre-purchase games’* |  |  |  |
| I8 Other |  |  | Home-join  Games – *‘you know that feeling…’*  Jackpots – *complex game variables* |  | Mobile – *play when you want, play on the go* | Home p4 – *on mobile or tablet*  Home-Promotions-Bingo – *mobile tablet* | Our bingo games – ‘Fill the gap while you wait’.  Mini games – ‘cure the itch’ withdrawal  Super Free Bingo – ‘you will also have the option to upgrade Free Bingo tickets to Superbooks tickets and win bigger prizes!’ | Our bingo games – ‘Fill the gap while you wait’. | Homepage – *play mirrorbingo on your mobile!* |  |  | Home *– app, tombola on the go*  Bingo games *– ‘bingo lite’, ‘the lightest of all’ – ties in with themes of safety and reassurance?*  Promotions – *on the go app*  Mobile app  Other games |
|  |  |  |  |  |  |  |  |  |  |  |  |  |
| **J. Tie-ins** |  |  |  |  |  |  |  |  |  |  |  |  |
| J1 News and current events eg. Royal wedding |  |  | Blog |  |  |  |  |  |  |  |  |  |
| J2 TV shows and films | Homepage – *I'm a Celebrity GMOOH*  Games – bingo slots – *The Osbournes reality TV* |  |  | Games slots – *The Osbournes reality TV* | Home  Promotions  Community-Magazine | Home p2  Home p3  Home-login/play now | Top Games – Deal or no deal | Bingo slots – Britain’s got talent, Deal or no Deal |  |  | Scratchcards – *‘Deal or No Deal’* |  |
| J3 Characters (comics, films, games) | Games – bingo slots – *Tomb Raider/Lara Croft* |  |  | Games slots – *Tomb Raider/Lara Croft* |  |  | Top games – Alice in Wonderland | Bingo slots – Beowulf, Alice in Wonderland |  |  |  |  |
| J4 Sport |  |  | Blog |  | Home-Family-RFLMuM  Promotions |  |  |  |  |  |  |  |
| J5 Other |  |  |  |  | Promotions  Community-Magazine |  | TV Advertisements | Bingo slots – Madness  TV Advertisements |  |  |  | Home – *Valentine’s Day special tie-in*  Promotions – *Valentine’s Day tie-in* |
|  |  |  |  |  |  |  |  |  |  |  |  |  |
| **K. Other** |  |  | Blog – *social media tie-in*  Games – casino games |  | Promotions – *playing on fear of missing out*  Mobile *– ‘no more missing out’*  Home-Bingo *– ‘miss out’*  Home-Responsible – *Gibraltar*  Community-Followme – *social media* |  | Customer support |  |  |  |  | Community 1 *– politically correct rules* |
